# Maternal Supply of Ruminally-Protected Lysine and Methionine During Close-Up Period Enhances Immunity and Growth Rate of Neonatal Calves

**DOI:** 10.3389/fvets.2021.780731

**Published:** 2021-12-02

**Authors:** Han Wang, Samy A. Elsaadawy, Zhaohai Wu, Dengpan P. Bu

**Affiliations:** ^1^State Key Laboratory of Animal Nutrition, Institute of Animal Science, Chinese Academy of Agricultural Sciences, Beijing, China; ^2^Joint Laboratory on Integrated Crop-Tree-Livestock Systems of the Chinese Academy of Agricultural Sciences (CAAS), Ethiopian Institute of Agricultural Research (EIAR) and World Agroforestry Centre (ICRAF), Beijing, China; ^3^Hunan Co-Innovation Center of Safety Animal Production, Changsha, China

**Keywords:** colostrum, ruminally protected amino acids, immunoglobulin, passive immunity transfer, transition cows

## Abstract

The objective of this study was to evaluate the effect of supplying ruminally-protected lysine (RPL), methionine (RPM), or the two in combination (RPML) to transition dairy cows on the immunity and performance of their offspring. Eighty heifer calves (*n* = 20 calves per group) were assigned to four treatments based on their dam diet; basal diet (CON), a basal diet with lysine [RPL, 0.33% of dry matter (DM)], a basal diet with methionine (RPM, 0.16% DM), or with the combination (RPML). Calves were fed colostrum from their dams within 2 h of birth. Calves were then fed milk only (d 2–22), a combination of milk and milk replacer (d 23–25), and milk replacer (d 25–60). Starter feed was fed to the calves twice daily after liquid feeding. Calves blood samples were collected after calving on 0, 12, 24, and 48 h and 5 and 7 d after birth. Data were analyzed by SAS software v9.4. Providing ruminally-protected amino acids (RPAA) to transition cows improved colostrum quality compared to the CON (Brix; *P* < 0.01). Serum total protein concentrations were higher in calves from supplemented cows than in calves from unsupplemented cows (*P* < 0.01). Calves born to dams in the RPM, RPL, and RPML groups had higher plasma immunoglobulin G (IgG) concentrations 0, 12, 24, and 48 h and 7 d after birth than those born to dams in the CON group (*P* < 0.05). The percentage of calves with adequate passive immunity transfer was increased with RPM and RPL or the two in combination (*P* < 0.01). However, there was no difference in the percentage of calves with adequate passive immunity transfer between the RPM and RPL groups (*P* = 0.21). Calves from cows that receive supplemental RPAA have a greater average daily gain (ADG) than those born to cows in the CON group (*P* < 0.01). These results indicate that maternal supplementation with RPM or RPL or the two in combination during the periparturient period could be an alternative strategy to improve the performance of calves, especially in accelerated growth programs in calves.

## Introduction

The transition period is a critical time when significant metabolic, physiological, and immunological changes occur for cows (1), as well as for their offspring (2). Nutritional supplementation strategies during the last trimester of gestation improve the performance of cows and their offspring (3, 4). Methionine (Met) and lysine (Lys) are considered limiting amino acids (AAs) in many lactating cow diets (5–7), and this also applies to calf diets (8). Maintaining levels of required AAs, especially Met and Lys, is very important for supporting normal fetal growth and subsequent milk production (9, 10). Maternal post-ruminal Met supply can also affect immune functioning in neonatal calves (11–13).

Calf management practices influence future lifetime performance (14). Therefore, colostrum management is very important, as neonatal calves are incapable of mounting an efficient and successful immune response at birth (15). Provision of adequate immunoglobulin (Ig) mass to calves is essential for survival, health, and future productivity (16). Colostrum components also participate in regulating the development of the intestinal mucosal immune barrier in newborn lambs during the passive immune transfer period (17). However, failed passive transfer (FPT) is a common problem in newborn calves (18), causing increased mortality, diarrhea, respiratory diseases, and septicemia in young calves (19–21), and even decreasing their first lactation milk yield (22, 23). For example, 46.5% of calf deaths during the pre-weaning and weaning periods are caused by pneumonia, with an approximate cost of $15 per calf per year (23, 24). Therefore, ensuring access to sufficient high-quality colostrum during the first few hours of life is essential to guarantee the adequate transfer of passive immunity to young calves.

Improving immune functioning can decrease health problems in calves (25), thereby improving productivity. All necessary immune components are present in neonates due to the development of immune defense mechanisms, which start in the uterus (15). Additionally, the placenta plays a central role in programming adult health and disease (26). Environmental stimuli, such as maternal nutrition, can cause changes in the supply of nutrients to the fetus (27). Thus, maternal feeding is fundamental to ensure offspring health and performance (28), as well as whole life performance. For example, Alharthi et al. (29) observed that calves born to cows fed ruminally protected Met (RPM) had higher body weight (BW) than calves from cows fed a control diet.

Research on fetal programming has been conducted on dairy cattle (30), sheep (31, 32), and non-ruminants (33–35), with a particular focus on nutrient manipulation during late pregnancy and its impact on the fetus and postnatal development of the offspring. Therefore, it was hypothesized that maternal supplementation of late gestation dairy cows with ruminally protected Lys (RPL), RPM, or the two in combination would, in addition to benefitting the cows themselves, increase the supply of metabolizable Met and Lys and other essential AAs that reach the calf through uteroplacental transfer, thereby aiding fetal development, contributing to increased bovine colostrum IgG, maximizing passive immunity transfer from cow to calf *via* colostrum, and enhancing the health status of calves. The current study aimed to evaluate the influence of maternal RPM and RPL supplementation on close-up dairy cows' colostrum quality, passive antibody transfer through the colostrum, and growth performance in their newborn heifer calves.

The results have been presented in partial form during the 2021 Annual Meeting of American Dairy Science Association (ADSA), Abstract No# 245 “Supplementing ruminally protected methionine or lysine improved milk production in transition cows” (36).

## Materials and Methods

### Experimental Design and Animals

This research is part of a large project to study the effect of ruminally protected amino acids on transition dairy cows. Cows management, sampling, analysis procedures and results were previously reported (36). Briefly, a total of 120 multiparous Holstein cows were distributed into eight groups (*n* = 15/replicate). Four treatments (*n* = 30 per treatment group) were given a basal diet without ruminally protected AAs (CON, *n* = 30) or the basal diet plus either RPM (*n* = 30), RPL (*n* = 30), or the two in combination (RPML, *n* = 30). The study was conducted at Shandong dairy farm, Shandong, China. Cows were selected based on days of pregnancy (250 ± 2 d), previous 305-d milk yield (11,512 ± 1,837 kg), parity (3.09 ± 1.56), and body condition score (BCS, 3.58 ± 0.26). The cows were fed diets from 21 d (25.0 ± 3.31 d) prior to the expected calving until 21 d (24.0 ± 3.31 d) post-calving. Feeding, ration formulation, chemical analyses, and essential AA profiles were described in detail (36).

Eighty Holstein heifer calves were assigned to groups based on the pre-calving treatment of their dam (CON, *n* = 20; RPM, *n* = 20; RPL, *n* = 20; or RPML, *n* = 20). The experiment was conducted as a completely randomized block design with treatments arranged in a 2 × 2 factorial. For all calves, the parturition process was continuously observed by veterinarians and technicians and *via* live cameras installed in the maternity barns. Calves were immediately separated from their dams after parturition and were not allowed to nurse. Calves were placed in individual Calf-Tel hutches (2.2 × 1.2 × 1.3 m; Hampel Corp., Germantown, WI, USA). The hutches were bedded with sand and placed on a sand base. Gave calves 4 liters (L) of colostrum within 2 hours (h) of birth *via* an esophageal feeder, followed by two more feedings of colostrum 6 h (3 L) and finally at 18 h (1 L); therefore, each calf consumed 8 L colostrum in total. Calves were dependent on colostrum feeding during the first day of life. In this study, female calves were only used, as we focus on milk production, and male calves are directly sold for fattening at the beef farms.

The dam of each calf was milked immediately after parturition, and gave each calf the first colostrum obtained from only their dam after pasteurization (the colostrum pasteurization protocol is detailed below). Samples of the colostrum fed to each calf were analyzed to determine colostrum quality using the Brix method (where 1% Brix is equivalent to the refractive index of a solution of 1 g sucrose in 100 g solution) with a DD-3 Digital-Dairy Refractometer (MISCO, Solon, OH, USA). Fed the suckling calves only fresh pasteurized milk from 2 to 20 d of age. From 21 to 23 d of age, fed the calves a combination of milk and milk replacer (MR; Eurolac Blue, Putten, Netherlands), according to the following MR volumetric ratios: 75% of milk and 25% MR at day 23 of age, then 50% milk and 50% MR at d 24 of age, and lastly 25% milk and 75% MR at d 25 of life. All neonatal calves were fed MR only from d 25 to 60 of age. In some cases (as in CON group), continued feeding MR even after 60 d until the calves reached to the required weaning weight, which is within 90 kg. The MR was dissolved in water to a final total solid content of about 17.90%.

The pelleted starter was fed to the calves twice daily from 4 d of age onward, after the morning and afternoon suckling (Rubeiyou 8100, Yuan Xing Co., Ltd., China). The composition and chemical analyses of the starter feed are presented in (Table 1). All calves were fed liquid feed (milk, MR, or a mixture of both) twice daily at 06:00 and 16:00 h using individual open buckets. The daily milk and MR quantities are presented in Table 2. Chemical analysis of milk and milk replacer (Table 3). When the starter ort was lower than ~30 g/day, added an additional 100 g starter to the following day to ensure that an adequate amount of starter was available at all times. Heifer calves were first weighed at birth prior to colostrum administration, then a second time at weaning to calculate average daily gain (ADG) using a HiWeigh Weighing System & Solution (model ACC2000, Suzhou, China). Heifer calves were weaned when their pellet feed intake reached 1.2 kg/d for 3 consecutive days. In addition to the weaning age (average 60 d), weaning calves' standards for body weight, height, and health were also considered before weaning. Therefore, the decision to wean the calves was a comprehensive process.

**Table 1:** Ingredients and chemical composition of pellet starter fed to Holstein heifer calves during the suckling period from birth to 60 days after calving.

**Items**	**(DM %)**
**Ingredients**	
Wheat bran	5.82
Steam-flaked corn	41.61
Cane molasses	1.62
Soybean meal	19.63
Extruded soybean	6.04
Canola meal	11.69
Corn gluten	2.48
Wheat shorts	7.11
Calves starter premix^a^	3.98
**Chemical analysis of nutrients**	
Dry matter, DM	98.25
Crude protein, CP	21.94
Ether extract, EE	4.01
Ash	7.47
Neutral detergent fiber, NDF	19.03
Acid detergent fiber, ADF	13.00
Calcium (Ca)	0.97
Phosphorus (P)	0.64

a *Amounts provided per kg of starter were as follows: vitamin A, 13,050 IU; vitamin D, 3,262 IU; vitamin E, 260.998 IU; Fe, 116.827 mg; Cu, 19.631 mg; Mn, 48.525 mg; Zn, 74.623 mg; Se, 0.766 mg; I, 1.342 mg; Co, 0.956 mg*.

**Table 2:** The daily amount of milk and milk replacer (MR) fed to Holstein heifer calves during the sucking period from birth to 60 days after calving.

**Age/day**	**Feed type**	**Milk amount kg/d**	**Remarks**
0 1 d old	Colostrum	4 + 3 + 1 L, 6 h apart	Colostrum can be enough to add 3 L
2 14 d old	Milk only	4 L/2 times	Gradually increase milk volume
15 25 d old	15–22 Milk only d 23, Milk 75 + 25%MR d 24, Milk 50 + 50%MR d 25, Milk 25 + 75%MR	5 L/2 times	15 18 days transition period
26 46 d old	Milk replacer only	6 L/2 times	26 29 days transition period
46 60 d old	Milk replacer only	3 L/2 times	0.7 1 L per day
60 d weaning	Milk replacer only	0	Feeding pellets

**Table 3:** Chemical analysis of milk and milk replacer (MR) fed to Holstein heifer calves during the suckling period from birth to 60 days after calving^a^.

**Items**	**Milk**	**Milk replacer**
DM, %	12.32	–
Density, g/L	1030.51	–
Milk protein, %	3.51	–
Milk fat, %	3.89	–
Lactose, %	4.37	–
Total solid, %	12.94	–
Dry matter, %	–	96.07
Crude protein, %	–	22.49
Ether extract, %	–	9.34
Neutral detergent fiber, %	–	0.77
Acid detergent fiber, %	–	0.55
Ash, %	–	7.15
Calcium (Ca), %	–	1.16
Phosphorus (P), %	–	0.98

a *Heifer calves were assigned to treatment groups based on the pre-calving treatment of their dam, receiving either an unsupplemented diet (CON) or a diet supplemented with ruminally-protected lysine (RPL) or methionine (RPM) or the two in combination (RPML)*.

### Ration Formulation

The detailed ration formulation protocol was presented previously (36). Briefly, the close-up diet was formulated using the Cornell Net Carbohydrate and Protein System [(CNCPS) v.6.5.5], which AMTS has implemented [AMTS Cattle. Professional v.4.7.2 (2016, AMTS LLC, Groton, NY, USA)] to meet or exceed the nutrient requirements for a close-up cow weighing 680 kg with a projected dry matter intake (DMI) of ~12.8 kg/d. Balanced Met and Lys concentrations according to the recommendations of CNCPS (v6.5) to be within 6.9% metabolizable protein (MP) Lys and 2.3% MP-Met. Ruminally-protected AA were first mixed with the premix then added to the total mixed ration (TMR) using a Vertical Feed Mixer (Supreme International Limited, Dodge City, Kansas, USA). The inclusion rate was 0.16% DM for RPM and 0.33% DM for RPL during the close-up pre-calving period.

The isopropyl ester of 2-hydroxy-4-(methylthio)-butanoic acid (HMBi) was supplied as a dry powder (Meta Smart Dry, Adisseo, France). According to the manufacturer, the product contains 57% HMBi, which is the equivalent of 78% Met, and has a ruminally absorbed of 50%. Thus, each gram of product provided 0.22 g metabolizable Met. RPL was supplied as a dry powder containing 47.5% L-Lys monohydrochloride (3.2.3) with 70% bioavailability, according to the manufacturer (LysiPEARL, Kemin Industries, Verona, MS, USA). Therefore, each gram of product provided 0.33 g metabolizable Lys-HCl.

### Feed and Liquid Feed

For calves, feed starter and MR samples were collected weekly, and amounts of dry matter (DM), crude protein (CP), ash, ether extract (EE), neutral detergent fiber (NDF), acid detergent fiber (ADF), calcium (Ca), and phosphorus (P) were determined as previously described (37). The concentrations of protein, fat, lactose, total solids and solids not-fat in milk were analyzed using mid-infrared procedures (MilkoScan FT3; Foss-600, Foss Analytics, Hillerød, Denmark).

For cows, the amounts of offered TMR and feed refusals were measured daily to calculate DMI for cows. Samples of TMR and major ingredients were collected and analyzed weekly. Details on feed sampling, composition, and analysis are presented elsewhere (36).

### Blood Sampling and Measurements

Duplicate blood samples of ~15 mL were collected from the jugular vein of each heifer calf 0, 12, 24, and 48 h, and 5 and 7 d after birth. Samples were collected into evacuated tubes containing either clot activator or lithium heparin for serum and plasma, respectively (Jiangsu Kangjian Medical Apparatus Co., Ltd, China). Samples were then centrifuged at 3,000 × g for 15 min at 4°C for separation of serum and plasma, and the supernatants were stored at −20°C until further analysis. Plasma concentrations of IgG were determined using enzyme-linked immunosorbent assay kits (GR-77091, Shanghai Ketao Biotechnology Center, Shanghai, China).

Serum total protein concentrations were determined using a digital temperature-compensating refractometer (Model 300027, SPER Scientific Ltd., Scottsdale, Arizona, USA). Before testing each sample, the refractometer prism was cleaned and the refractometer was calibrated with distilled water.

### Colostrum Testing, Freezing, Preservation, and Thawing Procedures

#### Colostrum Detection

Testing equipment included a thermometer, colostrum measuring instrument (hydrometer or refractometer), and tube. Colostrum refers to the milk expressed by cows within 12 h of delivery. Each time colostrum was expressed (maximum 30 min) its quality was determined. Sensory measurements included color and consistency. Milk with a peculiar smell or containing blood was disposed of. A hydrometer is used to measure the colostrum quality at a temperature of 21–27°C; specific gravity of 1.052–1.140 (green, the best colostrum quality) or 1.030–1.051 (yellow). Samples with a specific gravity below 1.030 (red) did not qualify. Using a refractometer, values ≥25 were considered good quality, 20–24 was considered medium quality, and 16–19 was considered poor quality. Quality colostrum was frozen after the addition of 1.25 ml/L formaldehyde (36–40%) and the date and time were recorded. When fresh colostrum was immediately fed to calves, 1.25 ml/L formaldehyde was added.

#### Freezing, Preservation, and Thawing of Colostrum

After pasteurization, the colostrum was placed in a refrigeration tank for ~30 min to cool down to 2–4°C. Then, the amount for that day was removed and the remainder was placed in colostrum bags in a freezer. Finally, colostrum samples of both sufficient and insufficient quality were labeled and stored in separate refrigerators. Frozen colostrum was thawed in a refrigerator at 5°C 24 h before use. Samples were then placed in a water bath at 42°C constant temperature of hot water or 50–55°C to thaw to 40°C. The quality of the colostrum was regularly tested after thawing to ensure that every newborn calf consumed quality colostrum.

### Statistical Analysis

Plasma immunoglobulin G (IgG) concentrations were analyzed using a model containing Met, Lys (fixed effects), time (repeated fixed effect), and their interaction using the PROC MIXED procedure in SAS (v9.4, SAS Institute Inc., Cary, NC, USA). The MIXED statistical model used for analysis was as follows:


$$\begin{array}{l} Y_{\text{ijklm}}=\mu +L_{\text{i}}+M_{\text{j}}+LM_{\text{ij}}+B_{\text{k}}+A_{\text{ijkl}}+T_{\text{m}}+LT_{\text{im}}\\ \;+MT_{\text{jm}}+LMT_{\text{ijm}}+\;\varepsilon_{\text{ijklm}}, \end{array}$$

Where Y_ijklm_ was the dependent, continuous variable; μ was the overall mean; L_i_ was the fixed effect of Lys; M_j_ was the fixed effect of Met; LM_ij_ was the interaction effect of Lys and Met; B_k_ is the effect of the block (B = 1,., 16); A_ijkl_ was the random effect of the th calf within the ijth treatment and within the kth block (B = 1,., n_ijk_); T_m_ was the repeated, fixed effect of time (h or day); LT_im_ was the interaction effect of time and Lys; MT_jm_ was the interaction effect of time and Met; LMT_ijm_ was the interaction effect of Lys, Met, and time; and ε_ijklm_ was the residual error.

Growth performance, colostrum quality and serum total protein data were analyzed considering the main effects of RPM, and RPL, and interaction RPM, RPL, using the following SAS model:


$$\begin{array}{l} Y_{\text{ijkl}}\;=\;\mu +L_{\text{i}}+M_{\text{j}}+LM_{\text{ij}}+B_{\text{k}}+A_{\text{ijkl}}+\;\varepsilon_{\text{ijkl}}, \end{array}$$

Where Y_ijkl_ was the dependent, continuous variable; μ was the overall mean; L_i_ was the fixed effect of Lys; M_j_ was the fixed effect of Met; LM_ij_ was the interaction effect of Lys and Met; B_k_ is the effect of the block (B = 1,., 16); A_ijkl_ was the random effect of the th calf within the ijth treatment and within the kth block (l = 1,., n_ijk_); and ε_ijkl_ was the residual error.

The Fisher exact test was used to compare the proportion of calves in the RPM, RPL, and RPML treatment groups with serum total protein concentrations ≥5.2 g/L with the same proportion of calves in the CON group. For each group, linear regression was used to determine whether there was a linear association between serum total protein concentrations and colostrum concentrations. If a significant association was found, 95% prediction intervals were calculated for various colostrum concentrations, and the colostrum concentration for which the lower limit of the prediction interval for serum total protein concentration was 5,200 mg/dL was determined.

Statistical power analysis was conducted prior to the experiment using a two-tailed test with α = 0.05, power = 0.90, and effect size = 0.35. The total number of calves required was projected to be ~78 calves, with 19 calves per treatment group, using G–Power v.3.1.9.2 Software (38). A 6% difference between treatment means for most variables was expected to be found with a power of 90%. Least-square means were compared using the least significant difference (LSD), and statistical differences were declared significant at *P* ≤ 0.05. Tendencies were considered from *P* > 0.05 to *P* ≤ 0.10.

## Results

### Colostrum Quality

Maternal supply of AA significantly affected colostrum quality, with higher colostrum quality obtained from cows fed RPAA than from those fed the CON diet (24.7 vs. 21.5 Brix, *P* < 0.01) (Table 4). Colostrum quality of cows in the RPML group was higher than that of cows who received either RPM or RPL separately (*P* < 0.01). There was no difference in colostrum quality between cows in the RPM and RPL treatment groups (*P* > 0.10).

**Table 4:** Effect of maternal supply of ruminally protected lysine or methionine and combination to pre-calving Holstein dairy cows on the immunity efficiency of their heifer calves^1^.

**Variable**	**Maternal Treatment^1^**					***P-value***			
	**CON**	**RPM**	**RPL**	**RPML**	**SEM^2^**	**TRT**	**RPM^3^**	**RPL^4^**	**RPM × RPL^5^**
Colostrum quality	21.51^c^	24.60^ab^	24.10^b^	25.40^a^	0.83	<0.01	0.03	0.04	0.14
IgG mg/ml									
0 h	3.16^c^	3.72^b^	3.65^b^	4.28^a^	0.29	0.05	0.04	0.06	0.88
12 h	4.40^b^	5.88^a^	5.76^a^	6.07^a^	0.19	<0.01	<0.01	0.02	0.04
24 h	5.10^b^	6.65^a^	6.51^a^	7.05^a^	0.23	<0.01	<0.01	0.04	0.04
48 h	5.11^b^	6.16^a^	6.27^a^	6.45^a^	0.24	0.01	0.01	0.03	0.06
7 day	4.77^b^	5.86^a^	6.07^a^	6.25^a^	0.19	<0.01	0.01	<0.01	0.02
Overall mean IgG	4.53^c^	5.66^b^	5.65^b^	6.05^a^	1.25	<0.01	<0.01	<0.01	0.05
Serum total protein(TP, g/dL)	5.80^c^	6.30^b^	6.40^b^	6.60^a^	0.10	–	0.01	<0.01	0.18
No. (%) of adequate passive immunity (TP> 5.2 g/dL)	83.40^c^	93.30^b^	93.40^b^	96.70^a^	2.40	–	<0.01	<0.01	<0.01

a, b, c *Mean values with different superscripts in the same row are significantly different (P < 0.05)*.

1 *Heifer calves were assigned to treatment groups based on the pre-calving treatment of their dam, receiving either an unsupplemented diet (CON) or a diet supplemented with ruminally protected lysine (RPL) or methionine (RPM) or the two in combination (RPML)*.

2 *Standard error of the means of all treatments*.

3 *RPM, effect of ruminally protected methionine*.

4 *RPL, effect of ruminally protected lysine*.

5 *Interaction of RPM × RPL*.

### Concentrations of IgG and Total Protein

**Figure 1:**
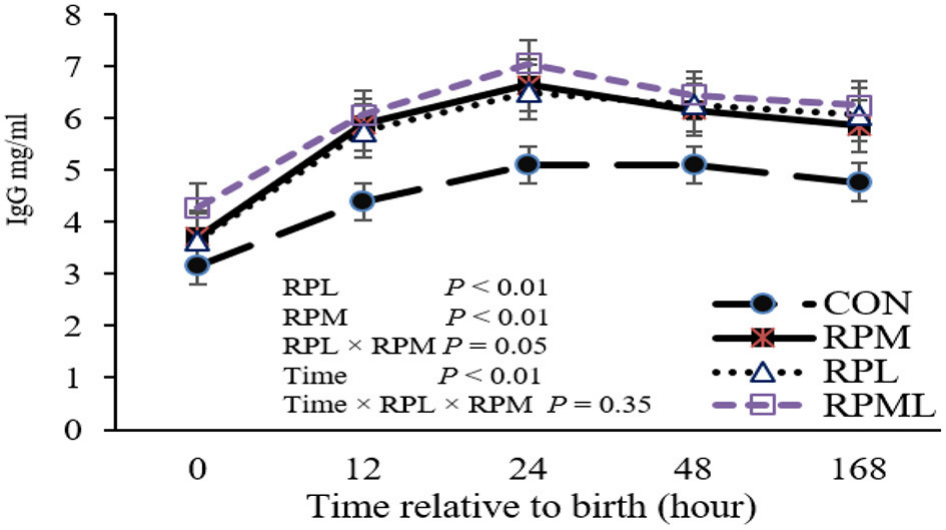
Effect of rumen-protected lysine and methionine supplementation to close-up cows on overall immunoglobulin G (IgG) concentrations of their heifer calves. Calves were assigned to treatments based on the pre-calving treatment of their dam, fed unsupplemented AA diet (CON), or receiving supplemental with ruminally-protected lysine (RPL) or methionine (RPM) or the two in combination (RPML). Values are means. Standard errors are represented by vertical bars.

Concentrations of plasma IgG at different time points are presented in Table 4. There was a significant effect of the maternal supply of AA on plasma IgG concentrations at birth (*P* < 0.05), with calves born to cows fed RPAA having greater plasma IgG concentrations than calves born to cows in the CON group. Maternal supply of RPM significantly affected IgG (*P* = 0.04), and RPL had a strong tendency to change IgG at calving (*P* = 0.06). Maternal supply of AA had an effect on plasma IgG at the remaining time points, as calves from RPAA groups had higher plasma IgG concentrations than those in the CON group at 12, 24, and 48 h and 7 d after calving (*P* < 0.05) (Table 4). There was a significant effect of time on plasma IgG concentrations (*P* < 0.01), with the highest IgG concentrations observed 24 h after calving (*P* < 0.01; 6.32 mg/ml) and the lowest IgG concentrations observed at birth (*P* < 0.001; 3.71 mg/ml), before colostrum administration. There was no difference between the RPM, RPL, and RPML groups at 12, 24, or 48 h or 7 d after calving (*P* < 0.05). None of the interactions between RPM × time, RPL × time, and RPM × RPL × time were significant (*P* < 0.10). There was a significant effect of the maternal supply of AA on overall plasma IgG concentrations (*P* < 0.05), with calves from cows that received AA supplementation having higher overall IgG concentrations than calves from cows that were fed the CON diet (4.53, 5.66, 5.65, and 6.05 mg/ml for CON, RPM, RPL, and RPML, respectively) (Figure 1).

Maternal supply of AA affected serum total protein concentrations on d 7 (*P* < 0.05), with calves from dams fed RPAA having higher serum total protein concentrations than those from dams fed the CON diet. Serum total protein concentrations of calves from cows fed RPML were also higher than those calves born to cows fed RPM or RPL separately (*P* < 0.01). However, there were no differences in total protein concentrations between calves whose dams were fed either RPM or RPL (*P* > 0.10) (Table 4).

The distribution of serum total protein concentrations for calves in the CON group was inclined. The percentage of calves with adequate passive immunity (total protein > 5.2 g/dL) was higher in the RPAA group than in the CON group (96.7 vs. 83.4%, *P* < 0.01), but there was no difference inadequate passive immunity between the RPM and RPL groups (*P* > 0.10). A greater percentage of calves born to cows fed RPML had adequate passive immunity than calves born to dams that were fed either RPM or RPL separately (96.7 vs. 93.4%, *P* < 0.01) (Table 4).

**Figure 2:**
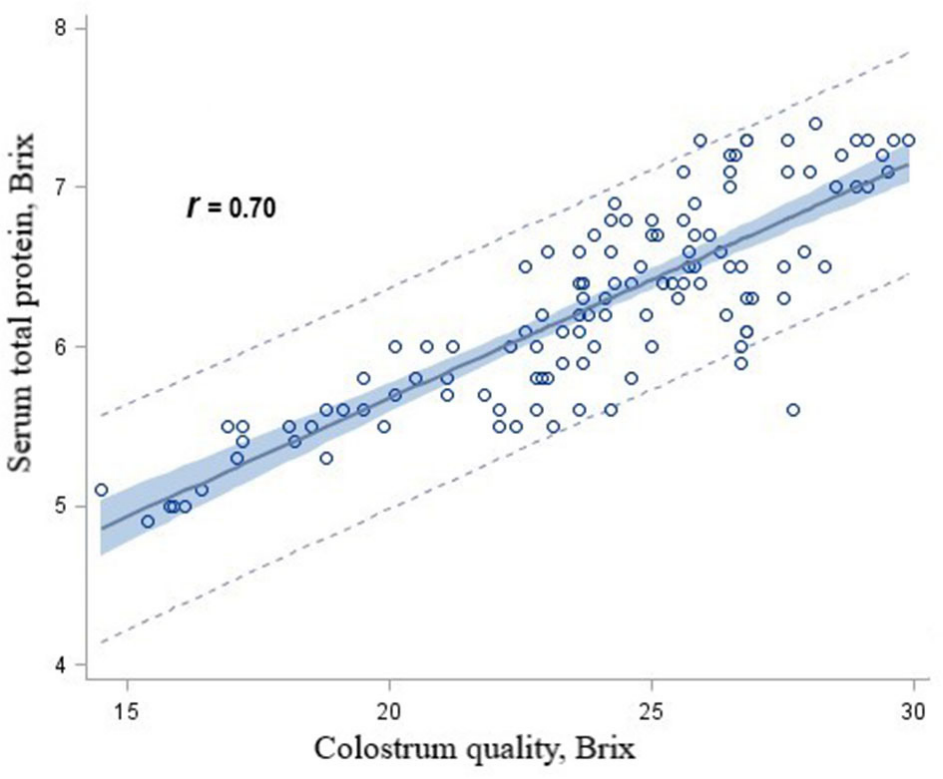
Scatterplot of serum total protein concentrations by colostrum quality overlaid with the fit line, a 95% confidence band and lower and upper 95% prediction limited for heifer calves born to Holstein dairy cows fed ruminally protected lysine or methionine or the two in combination during the close-up pre-calving period.

There were significant linear associations among serum total protein concentrations and colostrum concentrations for heifer calves in the CON (*r* = 0.78), RPM (*r* = 0.79), RPL (*r* = 0.72), and RPML (*r* = 0.86) groups. When data for calves from AA-supplemented cows were combined and 95% prediction intervals were calculated for various protein concentrations, it was determined that calves fed the RPM, RPL, or RPML diets received colostrum concentrations >21 Brix had serum total protein concentrations >5.2 g/dL at least 95% of the time (Figure 2).

### Growth Performance

The effects of the maternal supply of AA on growth efficiency are presented in Table 5. Calf birth weight was not affected by the maternal supply of RPAA to close-up cows (*P* > 0.10). Weaning age was significantly lower for calves from dams supplemented with RPAA than for those from dams in the CON group (*P* < 0.01). The weaning weights of calves from cows fed the RPML diet were higher than those calves born from cows in the other groups (*P* < 0.01). There was no difference in weaning weight between the RPM and RPL groups (*P* > 0.10) (Table 5).

**Table 5:** Effect of maternal supply of ruminally protected lysine or methionine and combination to pre-calving Holstein dairy cows on the growth performance of their heifer calves^1^.

**Variable**	**Maternal treatment^1^**					***P value***		
	**CON**	**RPM**	**RPL**	**RPML**	**SEM^2^**	**RPM^3^**	**RPL^4^**	**RPM × RPL^5^**
Birth weight^6^	38.60	38.60	38.20	38.50	0.63	0.79	0.76	0.81
Average daily gain kg/d	0.73^c^	0.85^b^	0.86^b^	0.91^a^	0.01	<0.01	<0.01	0.01
Total weight gain kg	50.20^b^	51.40^a^	51.30^b^	54.50^a^	0.73	0.02	0.04	0.18
Weaning weight kg	88.70^c^	90.00^b^	89.50^b^	93.10^a^	0.35	<0.01	<0.01	0.01
Age at weaning d	68.70^a^	60.00^b^	59.50^b^	59.80^b^	0.13	<0.01	<0.01	<0.01

a, b, c *italic>Mean values with different superscripts in the same row are significantly different (P < 0.05)*.

1 *Heifer calves were assigned to treatment groups based on the pre-calving treatment of their dam, receiving either an unsupplemented diet (CON) or a diet supplemented with ruminally protected lysine (RPL) or methionine (RPM) or the two in combination (RPML)*.

2 *Standard error of the means of all treatments*.

3 *RPM, effect of ruminally protected methionine*.

4 *RPL, effect of ruminally protected lysine*.

5 *Interaction of RPM × RPL*.

6 *Body weight at birth was measured directly after birth and before feeding colostrum*.

Mean total weight gain during the suckling period was significantly higher in calves from RPML cows than calves born to the other cows (*P* < 0.01). There was no difference in total weight gain between calves born to dams in the CON group and those born to dams in the RPM, RPL, or RPML groups (*P* > 0.10). The ADG of calves was improved by the maternal supply of RPAA during the pre-calving period (*P* < 0.01).

## Discussion

### Colostrum Quality

Colostrum quality was improved by maternal AA supply in the current study, and it is possible that the provision of RPM and RPL contributed to shifts in colostrum protein profiles, specifically affecting total serum solids (Brix) and plasma IgG. Colostrum management is key to calf rearing, health, and profitability, as newborns rely on both the immune- and nutrient-related qualities of colostrum (39). In the current study, stimulating DMI of cows fed AA during the close-up period resulted in greater energy and total metabolizable protein intake, which supported colostrum quality and was mainly attributed to increased efficiency of indispensable AAs, as discussed in our study (36). Radford et al. (40) reported that providing excess MP caused a shift in colostrum proteins, including immune regulators. Another reason explaining the improvement of colostrum in response to maternal AA supply in this study may be due to alterations in the uteroplacental transport of mechanistic target of rapamycin (mTOR) signaling, as previously reported (41), and essential and non-essential AA and glucose as well. It is potential that providing Lys and Met affect uteroplacental transport of nutrients *via* exact mechanisms. Lin et al. (42) indicated an increase in the mTOR downstream targets eukaryotic initiation factor 4E binding protein 1 and p70 ribosomal protein S6 kinase in due to feeding Lys.

Little is known about the minor proteins in colostrum, as the high concentrations of principal proteins reduce the detection sensitivity of mass spectrometry (43). However, supplying RPM to late gestation dairy cows (29) or beef cows did not affect colostrum quality (44), unlike the current results. The difference between our results and the results of Lievre (44) may be due to the difference of animals used in the experiment; in this study, all animals used are multiparous cows, unlike Lievre (44) where about 71 % of the animals are multiparous cows and about 28% are primiparous cows, as the quality of colostrum is greater in multiparous than primiparous cows.

### Concentrations of IgG and Total Protein

In the present study, higher serum total protein and IgG concentrations were observed for calves born to cows that were consumed RPM or RPL during the pre-calving period. The incorporation of Met and Lys may partially explain these results due to improving AA balance, stimulating colostrogenesis, and improve colostrum protein profiles, and increasing serum Brix and plasma IgG concentrations. Conversely, supplying RPM to prepartum beef cows or sheep decreased or did not affect concentrations of IgG and serum total protein (44, 45); likely that dietary do not provide adequate Met to support the immune system and the developing fetus in those studies (44, 45). Thus, the influence of RPM and RPL on colostrum quality and IgG absorption warrant further investigation.

Maternal supply of RPAA plays an important role and can have an effect on the immune system in newborn calves, as indicated by the higher concentration of serum Brix and IgG in claves conceived from supplemental-AA cows in the current study. These results similar to previous studies reported that pre-calving supply of RPM can influence immune function in the neonatal calf (11–13); three important antibodies of the immune system, IgA, IgG, and IgM, protect humans and animals, including calves, against many kinds of pathogens and viruses, activate the complement system, regulate antibody-dependent cell-mediated cytotoxicity, and improve immunity (46, 47). In addition, methyl-donors also act as anti-inflammatory regulators in ruminants, as increased methyl-donor (e.g., Met, betaine, choline, folate, and B12) supply in cows during the transition (48), and late gestation periods (12), leads to decreased haptoglobin, a biomarker used to predict disease.

The ingestion and absorption of sufficient quantities of IgG from colostrum promote the establishment of passive immunity in newborn calves. Therefore, the volume of colostrum consumed can also influence passive immune transfer (49). In the present study, the volume of colostrum given to calves was known, and because colostrum Brix concentrations differed between dietary treatments, it is reasonable to infer that RPM and RPL supplementation may have influenced IgG absorption as well as the quantity of IgG ingested by calves born to cows supplied with AA. Additionally, It has been shown that colostrum composition affects the absorption of IgG (50, 51). Thus, it is likely that differences in gastrointestinal development may account for the differences observed between calves born to cows supplemental with RPM and RPL and those born to non-supplemented cows in this study. Non-IgG constituents in colostrum may also affect the intestinal absorption of IgG.

Absorption of IgG *via* the intestinal epithelium is necessary to reduce calf morbidity and mortality, highlighting the importance of the development of the gastrointestinal tract *in utero* and uteroplacental nutrient transfer (52). Research in beef and dairy cattle is not readily available, but research on non-ruminant has shown that Met supply to late gestation swine alters the intestinal microbiota and gut morphology of offspring (35). Therefore, *via* similar mechanisms, maternal RPM, and RPL supply may possibly affect neonatal calves' gastrointestinal microbiota and gut morphology. Jacometo et al. (53) indicated that the fetus might utilize maternal Met. These authors also found that Holstein calves born to dams supplemented with RPM experienced faster gluconeogenesis and fatty acid oxidation in the liver (53), which may explain, in part, why calves born to cows received supplemental AA had greater ADG in the present study. In cattle, growth of the small intestine occurs twice as fast as whole-body fetal growth from d 175 to 280 of pregnancy (54). Several studies have reported the effects of maternal nutrition on gastrointestinal development in the fetus (35, 52). These findings provide evidence that improving the maternal supply of Met and Lys, and other essential AA, may cause changes related to the maturation of key biological pathways in the liver.

Serum total protein (Brix) is commonly used to indirectly measure IgG concentrations in dairy farming (18, 54, 55). In the current study, there was a strong relationship between colostrum Brix and serum Brix (*r* = 0.74). Measuring serum Brix concentrations using a highly specific and sensitive refractometer revealed a consistent association between IgG concentrations in calves in the first 7 d of life (18, 55). The correlation between serum IgG concentrations and serum total protein is relatively strong in calves; for example, Foster et al. (56) found linear associations between serum IgG and total protein concentrations in calves (*r* = 0.9). In the present study, the proportion of calves with adequate passive immunity did not differ between calves from dams in the RPM and RPL groups. However, <90% of calves in the CON group had adequate passive immunity in comparison with >93% of calves whose dams received AA supplementation. Therefore, our findings suggest that colostrum produced by cows that received AA supply would provide, in most cases, adequate passive immunity. Despite this, the influence of RPM and RPL on colostrum quality, IgG, and passive immunity requires further investigation.

### Passive Immunity

Transfer of passive immunity is usually considered adequate if serum IgG concentrations are >1,000 mg/dL in young calves fed colostrum (16). Results from this study indicated that the herd rate of failure passive transfer (FPT) was <10% for calves born to cows fed either RPM or RPL and only 3.3% for calves born to cows supplied with RPML, which is comparable to, or better than, rates previously reported for beef cows (57, 58). Failure of passive transfer occurs when calves fail to absorb a sufficient quality and/or quantity of IgG (44), which predisposes them to diseases (49); in the current study, it was minimal in calves that were fed colostrum from AA-supplemented cows. The recommended thresholds for healthy calves up to 8 d of life, which for serum protein concentrations is at least 5.2 g/dL, equating to 1 g/dL of serum IgG concentrations (59), were provided to the newborn calves *via* colostrum in the current study and tested colostrum Brix levels prior to administration.

Results from the present study demonstrated that the proportion of calves with FPT was higher in the CON group than calves born to cows consumed supplemental AA, indicating that FPT was minimized by AA supply. Research on late gestation beef cows (44) reported percentages of FPT around 5.2% when the animals were fed different levels of MP with or without RPM supply (44). However, the volume of colostrum given to calves in that study was likely unknown, as the newborn calves were left to suckle directly from their dams (44). Therefore, it was supposed that sufficient antibodies might not have been provided to the calves, leading to a higher incidence of FTP (44) compared to the present study.

In the current study, most diseased calves had serum total protein concentrations lower than 5.5 g/dL. It would be expected that calves with higher total protein concentrations would be less likely to develop various diseases, such as diarrhea and pneumonia. However, Tyler et al. (20) pointed out that there is little additional protection associated with concentrations substantially higher than threshold levels and noted that the risk of mortality among calves at calf-rearing facilities did not decrease as serum total protein concentrations increased above 5.5 g/dL. However, when other measures are used to identify calves with FPT, only those with FPT have an increased risk of death, and the risk of mortality does not decrease as serum IgG concentrations increase (22, 60). Although calf growth and future milk production are positively correlated with neonatal serum IgG concentrations (22, 60), the mechanism by which this occurs is unknown. Current recommendations are that 4 L high-quality colostrum with an average IgG concentration >50 g/L and total bacterial count <100,000 colony-forming units/mL be fed to calves during the first 6–8 h of life (16).

### Calf Growth Performance

Maternal AA supply during late gestation affects neonatal calf performance and immune responses (12, 53, 61), which agrees with the results from the current study. Nutrients in the diets of pregnant dairy cows are repartitioned toward colostrum production or fetal growth (62). Supplying RPM to pre-calving dairy cows affects fetal programming *via* changes in placental metabolism (63), and RPL may follow the exact mechanism. Similarly, supplying RPL to pre-calving cows likely affects metabolic and epigenetic signatures in the uteroplacental tissue, therefore, improving placental transport of nutrients to the fetus. However, the mechanisms involved and the role of Met and Lys in placental tissue is still unclear, underlining the importance of further work on assessing the effects of Met and Lys on the placenta.

In the present study, we observed a positive effect of maternal AA supply to pre-calving cows on calf growth performance, similar to previous studies on feeding RPM to close-up cows (29), but not in other (64). The results from this study may be due to alterations in the uteroplacental transport of essential and non-essential AAs and glucose and mechanistic target of rapamycin (mTOR) signaling, which has been indicated earlier (41). It is likely that supplying Met and Lys affects uteroplacental transport of nutrients through similar mechanisms. Lin et al. (42) reported an increase in the mTOR downstream targets eukaryotic initiation factor 4E binding protein 1 and p70 ribosomal protein S6 kinase in response to Lys supplementation. Besides uteroplacental transport, the results we observed in this study may have been due, in part, to alterations in colostrum composition, intestinal morphology interfering with IgG absorption or both.

Maternal nutrition can also influence the development of the intestinal tract in ruminants, which can affect the digestibility of nutrients and subsequent performance (32, 65, 66). RPM supply to late-gestation ewes increased both the protein expression of intestinal AA transporters and global methylation (32). Additionally, work on non-ruminants (35) showed that Met inclusion to late-gestation swine feeds altered the intestinal microbiota and gut morphology of their offspring. Therefore, it is possible that maternal RPM and RPL supply to close-up cows affect the gastrointestinal microbiota and gut morphology of their newborn calves *via* similar mechanisms. It remains to be determined whether the “fetal programming” effect of maternal Met and Lys has any direct effect on gastrointestinal development and/or nutrient oxidation. The silencing of glutaminase through promoter hypermethylation has been confirmed in colon cancer (67), and if such an effect occurs in response to dietary methyl-donors (e.g., Met, choline, and vitamin B12) it may alter the ability of this enzyme, which is abundant in gut intestinal cells, to utilize glutamine to generate glutamate (68).

In the present study, in addition to enhancing the colostrum quality by maternal AA supply to pre-calving cows, which may have been the main drivers of the increased ADG that was observed in calves born to AA-supplemented cows. Higher ADG values could be used as a marker of greater utilization by the developing gut (including the rumen epithelium) for oxidation (29). This is the first study to evaluate the effects of RPM and RPL supply in close-up dairy cows on the development of their heifer offspring.

## Conclusion

Supplementation of dairy cow diets with ruminally protected Lys or Met or the two in combination during the transition period enhanced the passive immunity, health, and growth rate of fetal-programmed calves as well as improved colostrum quality. These data demonstrate that maternal Met and Lys balance plays a crucial role in the fetal programming of calves. However, the mechanisms by which fetal programming improves performance require further research.

## Data Availability Statement

The original contributions presented in the study are included in the article/supplementary material, further inquiries can be directed to the corresponding author/s.

## Ethics Statement

All procedures were approved by the Animal Care and Use Committee of the Institute of Animal Science, Chinese Academy of Agricultural Sciences, Beijing, China (No. IAS20180115).

## Author Contributions

HW: formal analysis, writing – original draft, and investigation. SE: conducted the experiment, writing – original draft, and formal analysis. ZW: investigation. DB: conceptualization, review, editing, investigation, supervision, project administration, and funding acquisition. All authors contributed to the article and approved the submitted version.

## Funding

This research was partially supported by the Key Research and Development Program of the Ningxia Hui Autonomous Region (2021BEF02018), the International Atomic Energy Agency Technical Co-operation and Assistance Programme (No. CPR5025), the Agriculture Science and Technology Innovation Program (ASTIP-IAS07-1), Chinese Academy of Agricultural Science and Technology Innovation Project (CAAS-XTCX2016011-01), and Beijing Dairy Industry Innovation Team (BAIC06-2021).

## Conflict of Interest

The authors declare that the research was conducted in the absence of any commercial or financial relationships that could be construed as a potential conflict of interest.

## Publisher's Note

All claims expressed in this article are solely those of the authors and do not necessarily represent those of their affiliated organizations, or those of the publisher, the editors and the reviewers. Any product that may be evaluated in this article, or claim that may be made by its manufacturer, is not guaranteed or endorsed by the publisher.
